# Natural Products for Preventing and Managing Anthracycline-Induced Cardiotoxicity: A Comprehensive Review

**DOI:** 10.3390/cells13131151

**Published:** 2024-07-06

**Authors:** Jarosław Szponar, Przemysław Niziński, Jarosław Dudka, Kamila Kasprzak-Drozd, Anna Oniszczuk

**Affiliations:** 1 Clinical Department of Toxicology and Cardiology, Toxicology Clinic, Stefan Wyszyński Regional Specialist Hospital, Medical University of Lublin, 20-718 Lublin, Poland; jaroslaw.szponar@umlub.pl; 2 Department of Pharmacology, Medical University of Lublin, Radziwiłłowska 11 Street, 20-080 Lublin, Poland; przemyslaw.nizinski@umlub.pl; 3 Chair and Department of Toxicology, Medical University of Lublin, Jaczewskiego 8b, 20-090 Lublin, Poland; jaroslaw.dudka@umlub.pl; 4 Department of Inorganic Chemistry, Medical University of Lublin, Chodźki 4a, 20-093 Lublin, Poland; kamilakasprzakdrozd@umlub.pl

**Keywords:** cardiotoxicity, polyphenols, doxorubicin, cardio prevention, plant compounds

## Abstract

Doxorubicin (DOX) is an anthracycline anticancer agent that is highly effective in the treatment of solid tumors. Given the multiplicity of mechanisms involved in doxorubicin-induced cardiotoxicity, it is difficult to identify a precise molecular target for toxicity. The findings of a literature review suggest that natural products may offer cardioprotective benefits against doxorubicin-induced cardiotoxicity, both in vitro and in vivo. However, further confirmatory studies are required to substantiate this claim. It is of the utmost importance to direct greater attention towards the intricate signaling networks that are of paramount importance for the survival and dysfunction of cardiomyocytes. Notwithstanding encouraging progress made in preclinical studies of natural products for the prevention of DOX-induced cardiotoxicity, these have not yet been translated for clinical use. One of the most significant obstacles hindering the development of cardioprotective adjuvants based on natural products is the lack of adequate bioavailability in humans. This review presents an overview of current knowledge on doxorubicin DOX-induced cardiotoxicity, with a focus on the potential benefits of natural compounds and herbal preparations in preventing this adverse effect. As literature search engines, the browsers in the Scopus, PubMed, Web of Science databases and the ClinicalTrials.gov register were used.

## 1. Introduction

Cancer, a significant global public health concern, is the second leading cause of mortality after cardiovascular disease in humans [1]. The number of new cancer diagnoses is projected to increase by 70% over the next two decades [2]. There are numerous methods of treating cancer, with surgery being considered the most effective to date. Radiotherapy is a method of treatment utilizing ionizing radiation. Immunotherapy involves enhancement of the body’s immune response to treat cancer. This method allows destruction of cancer cells without damaging other body cells. Hormone therapy is a method of treating hormone-dependent cancers. This method is based on changing the hormonal environment, which inhibits the growth of this type of cancer. Hormone therapy and immunotherapy are not employed in the treatment of sarcomas. Chemotherapy is a pharmacological method of treating cancer. It utilizes cytostatic agents that affect the vital functions of cells. While new anticancer drugs have been developed and utilized in clinical settings, chemotherapy remains a primary treatment option for systemic and intermediate-stage metastatic cancers [3].

Chemotherapeutic drugs act as cytotoxic agents, which not only kill rapidly proliferating cancer cells, but also damage healthy ones, causing side effects. The latter may cause harm to cancer patients and reduce their quality of life [1]. In some cases, this can even result in death of the patient [2]. As such, these side effects limit drug dosage and patient compliance. It is thus evident that there is a pressing need for the discovery of chemotherapeutic drugs that are able to selectively attack cancer cells with minimal, if any, side effects and agents that are able to reduce the side effects of chemotherapeutic drugs. Some herbal medicines are known to be capable of alleviating complications caused by chemotherapy. For instance, PHY906, an extract derived from a combination of four herbs, has been shown to reduce chemotherapy-induced gastrointestinal toxicity [4].

Anthracyclines represent a group of chemotherapeutic agents that have proven efficacy in the treatment of solid tumors, particularly lymphomas and breast cancer, in patients of all ages. However, these agents have a major side effect in the form of cardiotoxicity, which can even affect the efficacy and safety of subsequent oncological treatments. Daunomycin was among the first anthracycline agents to be developed in the 1960s, and its association with heart disease was identified early on [5]. Doxorubicin (DOX) is an anthracycline anticancer agent that is commonly prescribed and highly effective in the treatment of solid tumors, including ovarian, breast, and gastrointestinal cancers, as well as hematological malignancies, such as lymphoma and childhood leukemia. Despite its clinical efficacy, DOX is limited by its cardiotoxicity, thus limiting its use in certain patient populations [1].

The three primary categories of anthracycline-induced cardiotoxicity (AIC) are early-onset, late-onset, and acute cardiotoxicity [5]. Early-onset AIC is defined as cardiotoxicity that occurs during the period of therapy or within one year following its conclusion. Late-onset AIC is defined as cardiotoxicity that occurs one year following the cessation of anthracycline treatment. Acute AIC manifests after a single dose, with clinical symptoms appearing approximately two weeks later. It is notable that the majority of cardiotoxicity occurs within the first year of anthracycline therapy. The cardiotoxicity of anthracyclines is primarily cumulative and dose-dependent, as evidenced by the well-documented and widely accepted mechanism of action, which involves the generation of reactive oxygen species (ROS) and subsequent formation of free radicals as a result of the electron–redox cycle of anthracyclines upon binding to DNA [6]. However, there is a growing body of evidence supporting the involvement of numerous additional mechanisms that have emerged in recent years. The recommendations for the follow-up of patients undergoing anthracycline therapy remain ambiguous. This ambiguity is attributable to two key factors. Firstly, the recommendations are either excessively restrictive, lacking sufficient evidence to justify such an approach, or excessively comprehensive, lacking clarity regarding the duration of patient follow-up. This flaw may inadvertently downplay the development of AIC in asymptomatic patients, as the aforementioned guidelines are mainly employed in symptomatic patients. Monitoring of anthracycline therapy is a crucial aspect in the prevention and treatment of anthracycline cardiotoxicity [7]. Consequently, researchers and clinicians must expeditiously identify more efficacious drugs to prevent DOX-induced cardiomyopathy and enhance the quality of life of individuals who have recovered from cancer.

This review presents an overview of current knowledge on DOX-induced cardiotoxicity, with a focus on the potential benefits of natural compounds and herbal preparations in preventing this adverse effect. As literature search engines, the browsers in the Scopus, PubMed, Web of Science databases and the ClinicalTrials.gov register were used. The following inquiries were used: “prevention and anthracycline-induced cardiotoxicity”, “polyphenols and anthracycline-induced cardiotoxicity”, “alkaloids and anthracycline-induced cardiotoxicity”, “saponins and anthracycline-induced cardiotoxicity”, “terpenoids and anthracycline-induced cardiotoxicity”, “polysaccharides and anthracycline-induced cardiotoxicity”, “natural products and anthracycline-induced cardiotoxicity”, “plant metabolites and anthracycline-induced cardiotoxicity”, “antracycline-induced cardiotoxicity and ‘in vitro’ studies”, “antracycline-induced cardiotoxicity and ‘clinical trials’”, “antracycline-induced cardiotoxicity and ‘animal models’”, “DOX and ‘in vitro’ studies”, “DOX and ‘clinical trials’”, “DOX and ‘animal models’”, “mechanisms and anthracycline-induced cardiotoxicity”, and “DOX and delivery methods”. Documents published from 1980 to April 2024 were included. In order to qualify work for the review the following exclusion criteria were applied: works that do not contain original data, e.g., reviews and comments; works which have not been independently peer-reviewed, e.g., conference papers, letters to editor, pre-prints etc.; works in languages other than English; papers published before 1980. 

## 2. What Patient Groups Are Considered to Be at High Risk for Cardiotoxicity?

**Figure 1:**
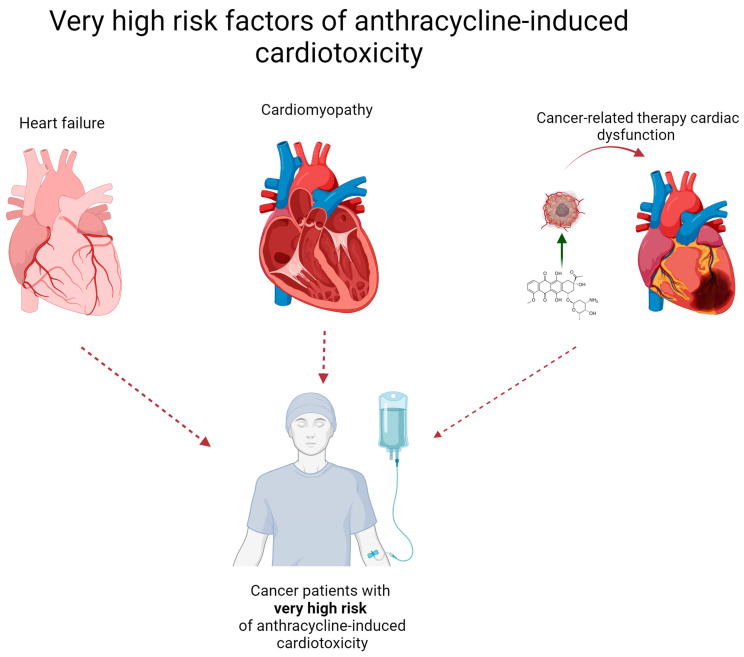
Very high-risk factors for anthracycline-induced cardiotoxicity [12].

**Figure 2:**
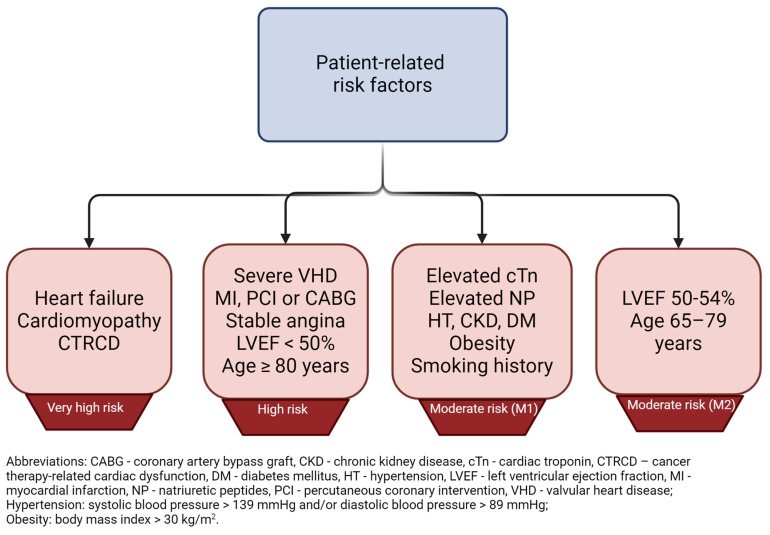
Patient-related risk factors including coexisting diseases, biomarkers, and lifestyle factors according to HFA/ICOS cardiovascular toxicity risk stratification (for anthracyclines only) [12].

**Figure 3:**
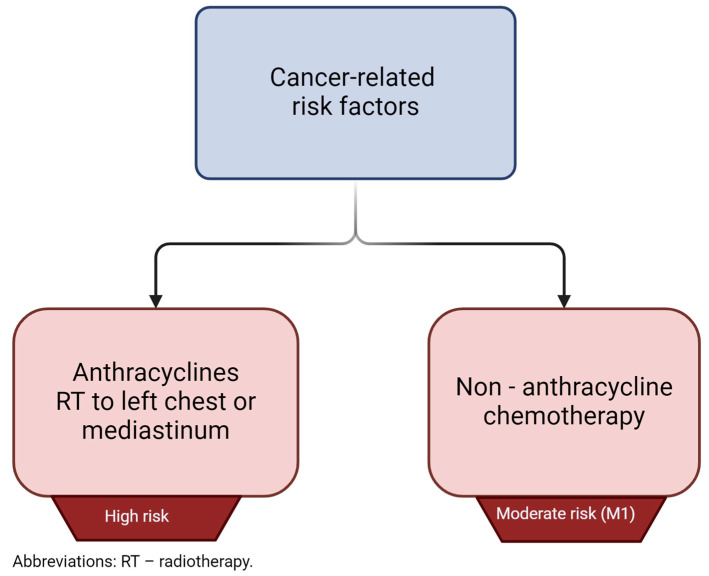
Cancer-related risk factors with regard to previous exposure to certain interventions according to HFA/ICOS cardiovascular toxicity risk stratification (for anthracyclines only) [12].

Anthracyclines are a relatively old group of anticancer agents, described in the late 1960s [8]. After more than fifty years from their introduction, anthracyclines are still widely used in the treatment of many types of tumors, and are also listed in the 23rd World Health Organization Model List of Essential Medicines, last published in 2023 [9]. Although the cardiovascular (CV) adverse effects (AEs) caused by anthracyclines were recognized shortly after their widespread clinical use, no clear and standardized definition of cardiotoxicity has been proposed [10]. Until recently, left ventricular (LV) dysfunction and heart failure (HF) were of the greatest concern; however, recent advances in cardio-oncology have led to many improvements in assessing the risk of anthracycline use and weighing the benefit-to-risk ratio [11]. In order to better manage cancer-related therapy cardiac dysfunction (CTRCD) by identification and classification of currently known risk factors, the European Society of Cardiology (ESC), in collaboration with other cardio-oncology societies, in 2022 developed and published comprehensive guidelines [12]. In general, ESC recommends CV toxicity risk assessment in all patients before starting potentially cardiotoxic anti-neoplasm therapy. The risk stratification method has been proposed and should be adopted prior to starting anthracycline administration [13]. When the probability of future risk of cardiovascular disease (CVD) is considered, patients can be divided into the following groups: low risk: <2%, medium risk: 2–9%, high risk: 10–19%, and very high risk: >20% [12]. To assess future risk and assign patients to certain groups, risk factors have been similarly classified, taking into account patient-related and cancer-related factors, with regard to severity of the symptoms. For instance, patients who already suffer from heart failure, cardiomyopathy, or any cancer therapy-related cardiac dysfunction are perceived as being at very high risk of anthracycline-induced cardiotoxicity. A graphical representation of very high-risk patients is shown in Figure 1. This score has been proposed by the Heart Failure Association (HFA) and International Cardio-Oncology Society (ICOS) and was implemented in the 2022 ESC guidelines on cardio-oncology [12]. The risk stratification score is the sum of both patient – related and cancer - related present risk factors and it has been calculated as follows: low risk = no risk factors or one moderate 1 risk factor; moderate risk (M) = moderate risk factors with a total of 2–4 points (moderate 1 [M1] = 1 point; moderate 2 [M2] = 2 points); high risk (H) = moderate risk factors with a total of ≥5 points OR any high-risk factor. For better visibility, patient-related and cancer-related risk factors are presented separately in Figure 2 and Figure 3, respectively.

The total planned dose of anthracyclines is also relevant in the context of risk assessment, due to the fact that the probability of CTRCD caused by anthracyclines is related with the total amount of the drug administered. The incidence of doxorubicin-induced left ventricular dysfunction increases from 3–5% to 18–48% when the cumulative administered dose rises from 400 mg/m^2^ to 700 mg/m^2^ [12]. The incidence of doxorubicin cardiomyopathy also grows dramatically along with the total dose, and it rises from about 4% when the dose is 500–550 mg/m^2^ to 36% when the dose exceeds 600 mg/m^2^ [14]. The most current recommendations suggest that ≥250 mg/m^2^ of doxorubicin or equivalent confer a higher risk of CTRCD [12]. The results of a multicenter cohort study by Feijen et al. provide evidence of the relations between selected anthracyclines and their potential to induce cardiotoxicity [15]. Based on data from about 28,000 participants, cardiomyopathy hazard ratios were calculated, and mean equivalence ratios for the most widely clinically anthracyclines were proposed. The mean values are based on the potential to induce CVD and do not take into account clinical efficacy. Doxorubicin was used as reference drug. The mean equivalence doses of selected anthracyclines are given in Table 1.

There is also growing evidence that genetic factors may play important role in CVD induced by anthracyclines. However, genetic testing is not a common practice before anthracycline administration due to the lack of proper recommendations; understanding and determining specific gene polymorphisms in anthracycline pharmacogenetics may be important in both preventive and therapeutic personalized medical approaches [12,16]. To date, more than sixty single nucleotide polymorphisms (SNPs) in about forty genes have been described [17]. In particular, the genes encoding ATP-binding cassette (ABC) and solute carrier (SLC) transporter are perceived as the most important in pharmacogenetic aspects of anthracycline-induced cardiotoxicity, since approximately 45% of all currently known SNPs are located in genes encoding drug transporters [18]. As whole-genome sequencing (WGS) techniques are becoming more accessible due to decreasing costs and increasing efficiency, they may provide very useful information on pharmacogenetic aspects of anthracycline-induced cardiotoxicity [19,20]. This comprehensive approach, when traditional and modern, personalized risk factors are taken into account, might help in more meaningful risk assessments, since preventive efforts may be engaged earlier and in a more effective way.

Furthermore, the use of other drugs by the patient has been found to have a significant impact on the development of cardiotoxicity. Recently, the cardiovascular safety of thiazolidinediones has been called into question. A study designed to assess the effect of two thiazolidinediones, rosiglitazone and pioglitazone, on DOX-induced cardiomyopathy in Wistar rats demonstrated that these drugs aggravate cardiomyopathy [21].

As is well documented, DOX treatment is associated with the development of cardiac and respiratory muscle pathology, which is attributed to the increased production of ROS, mitochondrial dysfunction, and impaired muscle contractility. Consequently, researchers have begun to examine hyperbaric oxygen therapy (HBO) with great interest. This method is regarded as controlled oxidative stress that can result in a significant and sustained increase in antioxidant expression in muscle. This HBO-induced increase in antioxidant capacity has the potential to improve the redox balance of cardiac and respiratory (i.e., diaphragm) muscle, preserving mitochondrial function and preventing muscle dysfunction. Nevertheless, the results obtained thus far in experimental animals indicate that modulating antioxidant expression in muscle by HBO therapy is not sufficient to prevent DOX-induced cardiorespiratory dysfunction [22].

## 3. Mechanisms of Anthracycline-Induced Cardiomyopathy

Anthracyclines may induce cardiotoxic effects at any stage of the treatment, yet the severity of the symptoms and prognosis of further heart disease are different and uncertain [23]. Anthracycline cardiotoxicity has been divided into three main types: 

1. acute toxicity: it develops immediately or up to two weeks after administration, is relatively rare (1% of the patients) and usually reversible, yet has the potential to evolve into the more chronic type [11];

2. early-onset chronic toxicity: develops within one year after anthracyclines were administered for the first time, is the most common (98% of patients), usually begins from asymptomatic left ventricular dysfunction that can evolve to symptomatic heart failure [11];

3. late-onset chronic toxicity: it can occur after many years, often even decades after treatment, and it is perceived as a late manifestation of early-onset toxicity with irreversible changes. It is frequent in adults who received anticancer anthracycline therapy in childhood and is the so-called Grinch syndrome [24].

Pajović et al. [25] conducted a study on two distinct phenotypes of doxorubicin-induced cardiomyopathy in rats, utilizing phenomapping for classification. The authors employed an experimental model of doxorubicin-induced cardiomyopathy to define phenotypes and associate them with changes in the cardiac transcriptome. The two major phenotypes exhibited comparably high mortality rates, with phenotype 1 displaying left ventricular (LV) dilatation, thinning of the LV posterior wall, a reduction in the LV ejection fraction (LVEF), and fractional shortening (LVFS). The two phenotypes were characterized by a number of different cardiac parameters. The first phenotype was associated with decreased baroreceptor effectiveness index (BEI) and increased NT-proBNP. The second phenotype was characterized by LV hypertrophy, with increased LV mass, preserved LVEF and LVFS, no changes in HRV and BEI, and a moderate NT-proBNP increase. The results of these studies indicate that both phenotypes occur with equal frequencies and have comparable adverse effects.

Despite the fact that it is not fully understood whether and what type of anthracycline-induced cardiotoxicity will develop, certain molecular mechanisms of anthracyclines’ impact on cardiac muscle and the cardiovascular system were investigated and described. Below, the main hypotheses are briefly reviewed. 

### 3.1. Oxidative Stress, Mitochondrial Dysfunction and Inflammatory Processes

Oxidative stress, caused by synthesis of an excessive amount of ROS in the cardiac tissue cells, is perceived as the main reason of cardiomyopathy induced by anthracyclines [26]. The majority of anthracyclines are reduced in cardiomyocytes to semiquinone, which can subsequently form quinone and superoxide (O_2_**˙**) [27]. Those redox reactions are mediated by intracellular enzymes such as endothelial nitric oxide synthase (eNOS) reductase and NADH reductase. O_2_**˙** free radicals undergo enzymatic catalysis by superoxide dismutase (SOD), resulting in hydrogen peroxide (H_2_O_2_), which can form, with the involvement of ferrous ions (Fe^2+^), the highly reactive hydroxyl radical (OH**˙**). Also, nitric oxide (NO), whose synthesis is increased by anthracyclines, may react with O_2_**˙,** resulting in the production of peroxynitrite anion (ONOO^−^), which is able to activate matrix metalloproteinase (MMP) [26,28,29]. Anthracyclines have an affinity with cardiolipin, which is a characteristic mitochondrial phospholipid. They can form an irreversible complex and cause impairment of the mitochondrial membrane, which is more susceptible to ROS and results in inhibition of the mitochondrial respiratory chain. Cardiolipin dysfunction promotes ROS upregulation, energy deficits, and even apoptosis via the p53 and caspase-3 signaling pathways [30]. Interestingly, p53-dependent mitochondria disfunction and caspase cascade activation can be mediated by macrophages. DOX can induce early recruitment of these immune cells and stimulate their secretion of epinephrine and norepinephrine, resulting in excessive activation of cardiac β-adrenergic receptors and promoting p53 nuclear effects [31].

ROS can be also formed as a result of alterations in iron metabolism in cardiac muscle cells. Anthracyclines are able to interact with iron regulatory proteins (IRPs) and ferritin (Ft), affecting iron homeostasis, thus increasing intracellular free iron levels and amplifying the oxidization processes catalyzed by iron [32]. Taking into account that cardiac tissue is sparse in antioxidant enzymes like catalase (CAT) [33] or SOD, it is highly vulnerable to ROS [29]. All of the abovementioned ROS have the ability to damage cellular structures, cause lipid peroxidation, and destroy proteins and nucleic acids, resulting in apoptosis and death of myocardial cells [34]. Anthracyclines can also induce inflammation, mainly via activation of the nucleotide-binding domain-like receptor protein 3 (NLRP3) inflammasome by toll-like receptor (TLR), nuclear factor kappa-light-chain-enhancer of activated B cells (NF-κB), and ROS. The activated inflammasome promotes secretion of pro-inflammatory cytokines, tumor necrosis factor-α (TNF-α), and interleukin-1β (IL-1β) and leads to inflammatory processes in the cardiomyocyte that can result in cardiotoxicity [35].

### 3.2. Calcium Levels Alterations, Apoptosis and Autophagy

Impaired calcium homeostasis is perceived as one of the key factors in the development of anthracycline-induced cardiotoxicity. Anthracyclines and their derivatives have the ability to disrupt calcium exchange-regulating genes and increase intracellular Ca^2+^ concentrations [26]. It has been reported that calcium ions can regulate the autophagy processes, which are crucial in maintaining cellular homeostasis and protecting the cell during stress circumstances [36]. Abnormally elevated Ca^2+^ levels in cardiomyocytes under oxidative stress conditions lead to impaired autophagy via dysregulation of the mTOR signaling pathway [37]. Alterations in certain elements’ (Ca, Fe) homeostasis, as well as oxidative stress and inflammatory processes in cardiomyocytes, may trigger programmed cell death pathways, including apoptosis, pyroptosis, and necroptosis. Ferroptosis is a relatively new phenomenon, which is caused by iron metabolism alterations in mitochondria induced by anthracyclines [38,39]. A detailed review concerning programmed cell death caused by anthracyclines was recently published [40], so readers are referred to this work in order to obtain more comprehensive information.

## 4. Brief Overview of the Most Significant Pharmaceutical Agents for the Prevention of Anthracycline-Induced Cardiotoxicity

### 4.1. Pharmacological Treatment

Anthracycline-induced cardiotoxicity can be prevented primarily by reducing the drug’s cardiotoxicity or using a less cardiotoxic derivative [41]. The use of a cardioprotective agent during treatment is a second option [42]. Medical therapies such as β-blockers, statins, angiotensin receptor blockers, angiotensin-converting enzyme inhibitors, aldosterone antagonists, sodium glucose transport protein 2 inhibitors, and dexrazoxane, as well as cardiorespiratory fitness, are cardioprotective strategies for either primary or secondary prevention.

β-blockers have been demonstrated to reduce left ventricular diameter, the risk of heart failure, and cardiomyocyte damage. Furthermore, they exert an influence on left ventricular systolic function [43]. This group of drugs exerts its effect by promoting neurohormonal regulation through the blockade of norepinephrine and epinephrine. This helps to reduce blood pressure and heart rate and improve heart function. In combination with angiotensin-converting enzyme inhibitors (ACEIs), they work in harmony to restore the LVEF. β-blockers have been shown to reduce mortality and morbidity in patients [44]. Carvedilol and nebivolol are examples of beta-blockers that are employed to counteract cardiotoxicity. Carvedilol has been demonstrated to possess antioxidant properties, which result in a reduction of ROS and mitochondrial damage. The effect of carvedilol is associated with a reduction in the expression of TLR4 and NF-*κ*B, whose signaling pathways has been implicated in carvedilol-induced apoptosis, as shown in an in vitro study [45]. Nebivolol exerts cardioprotective effects through stimulation of the β3 adrenergic receptor, which in turn activates endothelial nitric oxide synthase. β-blockers are particularly recommended for use in breast cancer patients. The protective effects of beta-blockers on heart failure were significant in breast cancer patients undergoing treatment with anthracycline and trastuzumab, with a 80% reduction in the risk of heart failure observed following the administration of β-blockers, which are particularly recommended for use in breast cancer patients. [46]. Furthermore, a placebo-controlled clinical trial demonstrated that carvedilol protects left ventricular function in anthracycline patients. Nevertheless, it is essential to monitor the dosage of beta-blockers in individual cancer patients to ensure their safety [47]. Moreover, to gain a full understanding of the direct mechanism, further long-term studies are required [48]. 

Statins such as atorvastatin have pleiotropic, anti-inflammatory, antioxidant, and lipid-lowering effects. AIC is largely attributed to oxidative stress in the heart. It is thought that cardioprotection is due to statins’ pleiotropic effects. Treatment with statins should be considered for primary prevention in adult patients with cancer at high and very-high cardiotoxicity risk [49]. The efficacy of angiotensin receptor blockers (ARBs) and angiotensin-converting enzyme inhibitors in preventing anthracycline-induced cardiotoxicity has been investigated in a number of studies. Blockade of the renin-angiotensin system has been demonstrated to be an effective method of preventing cardiotoxicity induced by doxorubicin [50]. A randomized controlled trial demonstrated that Valsartan (ARB) was an effective treatment for patients with non-Hodgkin lymphoma by preventing left ventricular dilation and QTc prolongation. Furthermore, a prospective cohort study in patients experiencing an acute myocardial infarction demonstrated that those on angiotensin converting enzyme inhibitors had better long-term survival outcomes than those on angiotensin receptor blockers [51]. In addition, angiotensin converting enzyme inhibitors with enalapril preserve cardiac morphology and function [52].

The results of trials investigating the cardioprotective effect of aldosterone antagonists have yielded divergent outcomes. While eplerenone demonstrated no cardioprotective effect in the chronic AIC mouse model [52], it did exhibit a restraining effect on doxorubicin in a second AIC mouse model [53]. This contradiction does not invalidate the impact of this group of medicines in the prevention of AIC, but highlights the need for further research to understand the mechanisms that underlie it. 

A reduction in the expression of inflammatory cytokines and an increase in cardiomyocyte viability were observed after administration of sodium glucose transport protein 2 (SGLT-2) inhibitors. [54]. In a separate study, doxorubicin in conjunction with empagliflozin was observed to result in a reduction in cardiac cell death and myocardial fibrosis [55]. In the year 2007, dexrazoxane became the first drug to be approved by the FDA for specific use in the treatment of anthracycline-induced cardiac injury. Dexrazoxane exerts its therapeutic effect by inhibition and depletion of topoisomerase II *β*. This results in protection of the heart. Dexrazoxane also inhibits oxygen radical formation through its action as an iron chelator, which binds free iron. Inhibition of free iron is the mechanism through which the cardioprotective effects of dexrazoxane are achieved [56]. The efficacy of dexrazoxane has been validated in pediatric clinical trials involving patients with cancer. In particular, dexrazoxane has been shown to be a valuable therapeutic option in pediatric patients with cancer who are at risk of developing cardiotoxicity associated with anthracycline chemotherapy [57].

**Figure 4:**
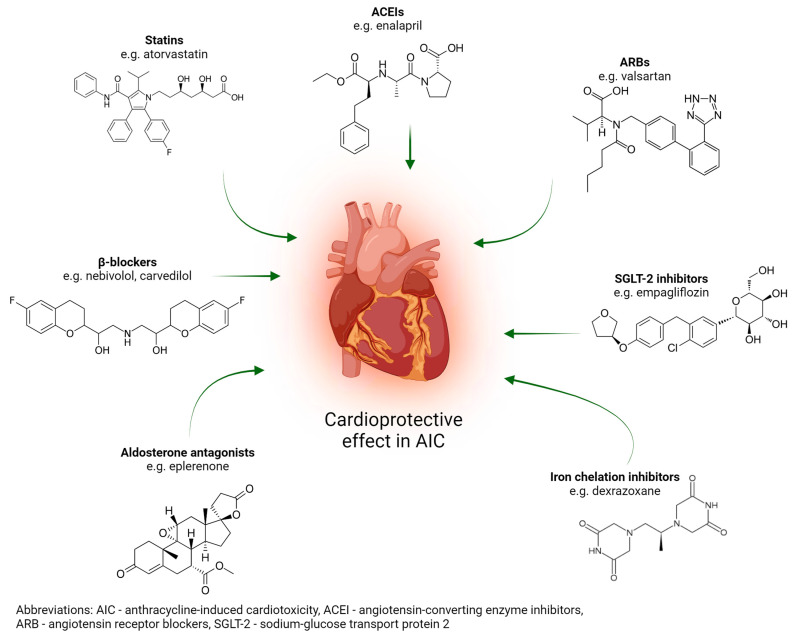
Selected pharmaceutical agents for the prevention of anthracycline-induced cardiotoxicity.

A schematic representation of selected pharmaceutical approaches alleviating AIC is presented in Figure 4.

A review of the literature reveals that the cardiorespiratory fitness of breast cancer patients is adversely affected by anthracycline chemotherapy. Conversely, aerobic exercise has been shown to stimulate the synthesis of myofilaments in different animal models, a phenomenon which is regarded as a positive outcome [58]. Therefore, it is of paramount importance to ensure that patients undergoing anthracycline therapy engage in physical, aerobic activity.

### 4.2. New DOX Delivery Methods and DOX Derivatives

With the development of formulation technology, a number of new drug delivery methods have been developed, such as liposomes, nanoparticles, micelles, and dendrimers, which can improve in vivo drug distribution, increase drug bioavailability and therapeutic index, and ensure high safety. To date, two types of liposomal DOX have been approved for clinical use, as follows: pegylated liposomal DOX (PLD, Doxil^®^/Caelyx^®^, Lipdox^®^) and non-PLD (NPLD, Myocet^®^) [59]. Liposomal DOX can reduce myocardial drug concentrations, attenuate the extent of myocardial DNA damage, and upregulate interferon-stimulated gene and translocator protein expression. This activates pro-survival signals and reduces cardiotoxicity [60]. Breast cancer patients treated with liposomal DOX have been shown to have significantly lower death rates [61].

Polymer micelles exhibit excellent stability, robust drug-carrying capacity, and the ability to enhance the solubility of drugs. Researchers have developed a light-responsive Poly-DOX-M polymer micelle, which was enriched in the tumor site through enhanced permeability and retention. This micelle was capable of releasing the drug efficiently after ultraviolet irradiation and demonstrated a significant impact on tumor inhibition. Histopathological analysis of heart, liver, and spleen tissue revealed that the polymer micelles did not cause any discernible tissue damage. The aforementioned evidence indicates that the polymer micelles effectively reduce the systemic toxicity of DOX and exhibit good biological safety [62].

Nanoparticle drug delivery has the characteristics of targeted drug delivery, prolonged circulation in the blood, and enhanced drug internalization, which can improve the clinical therapeutic effect of anticancer drugs [63]. Yang et al. introduced a trisulfide bond into the DOX homodimer prodrug to promote its self-organization into uniform, stable nanostructures [64]. This form exhibited very high sensitivity to reduced glutathione (GSH), enabling rapid drug release in tumor cells, greatly reducing DOX toxicity to normal cells and effectively avoiding damage to major organs including the heart, liver, and kidneys. Other researchers have developed an oral DOX nanomedicine that uses catechol-modified CS/hyaluronic acid nanoparticles as a carrier, and the drug carrier can better adhere to the oral mucosa and has the ability to deliver DOX continuously. Studies have shown that this oral formulation has better cell uptake and can inhibit the growth of oral squamous cell cancer [65]. However, in vivo experiments are still needed to further confirm the clinical effect of this nanomedicine.

Dendrimers are a novel class of macromolecules with highly branched and monodisperse characteristics. Their surface groups are readily amenable to modification, allowing them to be prepared as multifunctional drug carriers exhibiting excellent biocompatibility, tumor-targeting properties, and safety profiles [66]. Dendrimers can encapsulate DOX and subsequently release the drug in a targeted manner, while the resulting drug carrier exhibits characteristics such as reduced organ toxicity, controlled drug release, and high drug permeability [67].

A novel and highly promising approach to the prevention or reduction of anthracycline-induced cardiotoxicity is the development of novel anthracycline derivatives with minimal or no cardiotoxicity (Table 2).

## 5. Natural Drugs for Preventing Anthracycline-Induced Cardiomyopathy 

### 5.1. Polyphenols

Supporting data demonstrated that phytochemicals can reduce DOX-induced myocardial damage by reducing ROS and apoptosis and/or increasing the activities and/or protein expression of endogenous antioxidant enzymes in in vitro cell models and in vivo animal studies [1]. Polyphenols are organic compounds belonging to the phenol group. The name polyphenolic compounds signifies that there must be at least two such hydroxyl groups. They are most commonly found in edible plants and in the skins of fruits, where they occur at the highest concentration. Due to their chemical structure, they are able to act as antioxidants and free radical scavengers.

The results of studies have shown that quercetin is effective in protecting against doxorubicin-induced cardiomyopathy. This occurs through the increase in nuclear factor-erythroid 2-related factor 2 (Nrf2) expression, which alleviates LV dysfunction and decrease histological abnormalities [70]. The findings confirmed that quercetin, when combined with cyclophosphamide, activated the ERK1/2 signaling pathway in H9c2 cells, thereby inhibiting the metastasis of MDA-MB-231 cells, alleviating myocardial injury in cyclophosphamide-treated mice, and augmenting the efficacy of breast cancer chemotherapy [71]. Quercetin plus sitagliptin can significantly reduce lactate dehydrogenase (LDH), C-reactive protein, troponin, and creatine kinase (CK) levels compared to DOX rats treated with quercetin or sitagliptin alone. In addition, by reducing lipid concentrations and exerting antioxidant and anti-inflammatory effects, they alleviate DOX-induced cardiomyopathy [72].

Baicalin has been shown to reduce the dose of DOX in cancer treatment and to reduce dose-dependent cardiotoxicity. This is due to the fact that intake of the aforementioned substance leads to a reduction in mitochondrial membrane potential, which is brought about by the effect it has on the ROS/[Ca^2+^] pathway. This increases the sensitivity of breast cancer cells to DOX and induces cancer cell apoptosis [73]. In addition, baicalein can reduce ROS production and cell death and attenuate myocardial injury and histopathological damage, thereby inhibiting N-terminal c-JunJ kinase activation and reducing oxidative stress and myocardial apoptosis in BALB/c chick cardiomyocytes [74].

Zhang et al. [75] and Zare et al. [76] found lutein and apigenin to have antioxidative and antiapoptotic effects, reducing serum heart damage and apoptotic markers in DOX rats, reducing myocardium malondialdehyde levels, and increasing SOD levels. Luteolin has been demonstrated to inhibit phosphorylation of Phlpp1, regulate the AKT/Bcl-2 pathway, and inhibit apoptosis of cardiomyocytes [75]. In addition, in combination with DOX, it can further reduce tumor mass by inhibiting the activity of breast cancer cells [77]. Luteolin significantly increased LV strain and LVEF in DOX mice and attenuated DOX-induced heart failure. It was also shown to enhance mitophagy via the Drp1/mTOR/TFE pathway, significantly decrease DOX-induced ROS levels, enhance mitochondrial membrane potential in rat cardiac myocytes, and improve DOX-induced cardiac myocyte injury [78].

Epigallocatechin-3-gallate is a compound belonging to the flavonoid group, a polyphenol derived from plants, and a catechin derivative [79]. Green tea is a significant source of epigallocatechin-3-gallate, with levels reaching up to one-third of the dry matter. It has been shown to be effective in the reduction of myocardial damage and the protection of heart function. It reduces myocardial inflammation and oxidative stress by attenuating apoptosis. In addition, it has been observed to reverse intracellular Ca^2+^ depletion, attenuate reactive oxygen species, and increase the expression of MnSOD and the apoptotic pathway [80]. 

Research by Saleh et al. [81] has shown that catechin can reverse the decrease and increase in myocardial antioxidant enzyme levels induced by DOX, as well as reduce mitochondrial degeneration and apoptosis. Furthermore, it has been shown to decrease lipid peroxidation. The capacity of catechin to forestall DOX-induced cardiotoxicity in rats was evaluated by Ozyurt et al. [82]. The animals were administered intravenous DOX (at a dose of 3 mg/kg/week), intraperitoneal catechin (at a dose of 200 mg/kg/week), or catechin in combination with DOX. The experiments were conducted for one month. The administration of catechin at a dose of 20 mg/kg was observed to result in the most significant reduction in DOX cardiotoxicity. The data from preclinical studies indicate that catechins may prevent cardiotoxicity induced by DOX. However, further testing is required to ascertain whether they can be employed as an effective component of a chemotherapy-supporting diet for oncological patients. Another polyphenol, corilagin, has been demonstrated to improve the reduction in heart rate and blood pressure in rats following DOX administration. Additionally, it has been shown to inhibit the P13K/Akt/NF-κB pathway, thereby reducing inflammatory damage and apoptosis in myocardial tissue [83]. 

Resveratrol is a naturally occurring phytoalexin produced by certain higher plants. It is a derivative of stilbene, which is included in the group of plant polyphenols. The most abundant source of trans-resveratrol is Japanese knotweed. Furthermore, resveratrol is a valuable component of dark grape juice. It is also present in wine, particularly red wine. The search for new sources of this compound continues due to its unique properties. It has anti-inflammatory, antioxidant, anti-apoptotic, and cardioprotective properties [84]. Resveratrol has been shown to inhibit histone H2AX acetylation, ameliorate doxorubicin-induced cardiac dysfunction, and improve cardiomyocyte DNA damage repair. In DOX rats treated with resveratrol at 50 mg/kg for 6 weeks, resveratrol inhibited apoptosis and reduced cardiac injury [85]. The mechanism involved was to activate the VEGF-B/Akt/GSK-3β signaling pathway, thereby protecting cardiomyocytes from DOX toxicity. Resveratrol supplementation during pregnancy was found to improve the antioxidant system in young rat cardiomyocytes, reducing cell necrosis and oxidative DNA damage [86]. In addition, resveratrol has a cardioprotective effect on young DOX mice. The specific mechanism of action of this compound was to inhibit the NLRP3 inflammasome-mediated signaling pathway, thereby ameliorating the delayed hypertension-induced cardiomyopathy and reducing macrophage infiltration and inflammatory lesions in the heart [87]. It has also been shown that resveratrol can reduce NO levels in DOX rats, prevent DOX-induced myocardial apoptosis, and promote autophagy in vitro and in vivo [88]. Furthermore, resveratrol was found to reduce doxorubicin-induced myocardial apoptosis in mice through upregulation of sirtuin 1-mediated p53 deacetylation and induction of hemoxygenase and hemoxygenase-1 [89]. 

A study has demonstrated that ferulic acid possesses potent antioxidant activity. Oral administration of ferulic acid has been shown to significantly reduce B-type brain natriuretic peptide (BNP), creatine kinase-MB (CK-MB), LDH, IL-1β, and IL-6 levels in the serum of DOX rats. Furthermore, studies have shown that this compound can enhance the production of GSH, while also reducing oxidative stress and inflammatory responses. Additionally, ferulic acid can regulate blood pressure in a normalizing manner [90]. 

The objective of a study conducted by Maiuolo et al. [91] was to investigate the effects of these bergamot polyphenolic fractions, oleuropein, and cynara cardunculus extract on an in vitro model of cardiotoxicity induced by the treatment of rat embryonic cardiomyoblasts (H9c2) with doxorubicin. The study examined the biological mechanisms involved and the crosstalk existing between the mitochondria and the endoplasmic reticulum. The three aforementioned plant derivatives demonstrated the ability to mitigate the adverse effects caused by exposure to doxorubicin. It was found that these natural compounds had the effect of reducing cell mortality and oxidative damage, increasing the lipid content, and decreasing the concentration of calcium ions that escaped from the endoplasmic reticulum. A study conducted by Patil et al. [92] investigated the potential role of polyphenol-rich extracts derived from *T. cacao* beans as a means of conferring organ-protective properties in mice bearing Ehrlich ascites carcinoma and subjected to doxorubicin-induced toxicity. Treatment with cocoa bean extract resulted in a significant increase in survival time and life span percentage, as well as an enhancement in the antioxidant defense system. Furthermore, the extract markedly elevated biomarkers for cardiac, hepatic, and renal function, along with markers indicative of oxidative stress. Additionally, it mitigated the pathological alterations induced by doxorubicin. The cocoa bean extract demonstrated anti-organ-toxicity properties, as well as potent antioxidant and anticancer activities. Consequently, the cocoa bean extract may be employed as an adjuvant nutraceutical in cancer chemotherapy. A further study demonstrated that a smoothie containing a mixture of *Citrus sinensis* and *Vitis vinifera* L. cv. Aglianico N., which are typical Mediterranean diet fruits, possesses bioactive polyphenols that protect cardiomyocytes against the damaging effects of doxorubicin-induced oxidative stress. The authors demonstrated that the mixture of Citrus sinensis and Vitis vinifera in a 1:1 ratio was effective in reducing cardiomyocyte damage resulting from anthracyclines without significantly interfering with the pro-apoptotic activity of the drug on breast cancer cells. These findings suggest the potential use of vegetable smoothies as functional foods in the context of chemotherapeutic anticancer protocols [93]. The protective effects of other polyphenols on anthracycline-induced cardiotoxicity are presented in Table 3.

### 5.2. Alkaloids

Alkaloids are a group of naturally occurring basic organic compounds (generally heterocyclic), mainly of plant origin, containing nitrogen. The precursors for the biosynthesis of these compounds are amino acids. Alkaloids usually exhibit strong, sometimes toxic, physiological effects on the human body. From a physiological point of view, alkaloids act as secondary metabolites, protecting the plant from herbivores (by imparting a bitter taste), microorganisms, and infections.

Berberine is an alkaloid derived from a variety of natural sources, including barberry, grape, and oregano. This plant compound has been used in traditional Asian medicine for centuries and is increasingly being used in modern natural medicine. The results of a study indicated that berberine treatment led to a reduction in the ascites in DOX rats, accompanied by the restoration of CK, CK-MB, CAT, SOD, and lipid peroxidation marker (MDA) levels in the serum. Furthermore, the observed efficacy of this treatment included the effective alleviation of bleeding and necrosis of cardiac interstitial tissue, along with the reduction of inflammatory cell infiltration. Additionally, the demonstrated inhibitory effect of berberine on Ca^2+^ overload and reduction of mitochondrial membrane potential, in conjunction with the improvement of DOX-induced acute myocardial damage, further supports its therapeutic potential [101]. Experiments were conducted in vivo and in vitro with rats treated with berberine and DOX by Wu et al. [102]. The combined treatment of DOX and berberine was found to significantly enhance the survival rate of rats. Furthermore, it was observed that electrocardiogram abnormalities and tissue damage were both improved. Specifically, it was observed that berberine inhibited expression of p66Shc through the activation of sirtuin 1 (SIRT1), which resulted in the attenuation of oxidative stress and apoptosis, as well as the protection of cardiomyocytes from the damage caused by DOX. The findings of Zhao et al. [103] indicate that berberine improves survival rates and body weight while also reducing myocardial injury in BALB/c mice. Lv et al. [104] demonstrated in their study that the plant constituent inhibits mitochondrial dysfunction-mediated cardiomyocyte apoptosis and metabolism of doxorubicin. They also reduce the accumulation of doxorubicinol in rat cardiomyocytes. Matrine is a natural product belonging to the alkaloid class, exhibiting anti-inflammatory, immunomodulatory, antibacterial, and antioxidative properties. A study has shown that matrine can reverse the downregulation of UCP2 induced by DOX, which inhibits ROS production and apoptosis in cardiac tissue [105]. A study by Zhang et al. [106] showed that matrine regulates the RPS5/p38 signaling pathway, resulting in a notable reduction in the proliferation and metastasis of mouse cardiac fibroblasts. Moreover, the study found that matrine was able to improve pathological cardiac fibrosis and cardiac insufficiency.

### 5.3. Saponins

Saponins are chemical compounds that can be found in more than 500 plant species. Saponins are a group of glycosides that contain two main parts: an aglycone (called a sapogenin) and a sugar residue. This unique combination of properties and biological activities makes saponins an invaluable resource. Saponins have a number of applications in the pharmaceutical industry and in medicine, where they exhibit a range of medicinal properties, including anti-inflammatory, antimicrobial, and expectorant effects [5]. Ginsenosides are the primary biologically active components of ginseng, belonging to the group of triterpene saponins. They demonstrate cardioprotective and anti-tumor effects. 

In a study by Xu et al. [107], mice were administered ginsenoside Rg1 at a dosage of 50 mg/kg intraperitoneally for seven consecutive days in a DOX mouse model. This resulted in a reduction in the changes to ejection fraction and fraction shortening that would otherwise have been induced by DOX. Moreover, the plant constituent was observed to enhance endoplasmic reticulum stress and excessive autophagy in the mouse heart, as well as regulate the expression of transcription intermediary factor 1 (TIF1), glucose-regulated protein 78 (GRP78), and p70S6 kinase. The results indicated a notable reduction in DOX-induced cardiotoxicity, as evidenced by a decline in the observed toxicity. The efficacy of ginsenoside Rg1 in enhancing the anti-proliferative effect of DOX on MDA-MB-231 cells has been demonstrated. The presence of ginsenoside Rg1 resulted in a significant increase in reactive oxygen species and DNA damage in cancer cells. In addition, the ginsenoside Rg1 has been observed to reduce the level of mitogen-activated protein kinase (MAPK) gene expression, induce apoptosis, and display minimal toxicity towards MCF10A normal breast cancer cells [108]. Furthermore, research has shown that the ginsenoside Rh2 has beneficial effects on H9c2 cells in male Swiss mice. In particular, we have observed that it enhances cell viability, inhibits the release of cardiac enzymes, and improves pathological changes and apoptosis. These effects are likely responsible for the amelioration of decreased oxidant biomolecules and malondialdehyde [109]. Nevertheless, the ginsenoside Rg3 was observed to restore abnormal vascular function and myocardial function, promote cell viability, and thus attenuate oxidative damage and apoptosis. Furthermore, the compound was observed to activate the Nrf2-ARE pathway in cardiac microvascular endothelial cells derived from Sprague Dawley rats [110].

Astragaloside IV is an active compound derived from astragalus, a plant with a long history of use in Chinese and Korean folk medicine. Astragaloside IV has been demonstrated to inhibit NADPH oxidase 2 (NOX2) and NOX4-mediated oxidative stress in cardiomyocytes, thereby alleviating cardiomyopathy in DOX rats [111]. Furthermore, astragaloside IV was shown to participate in the Nrf2 signaling pathway, increasing the expression of GPx4. This has the effect of effectively reducing myocardial ferroptosis and exerting a protective effect on cardiomyopathy [111]. Chen et al. showed that salidroside could activate the AMPK signaling pathway, subsequently regulating cellular lipid metabolism, reducing iron accumulation, and enhancing GPx4-dependent antioxidant capacity. This resulted in the improvement of mitochondrial function and the significant inhibition of ferroptosis and fibrosis in DOX mouse cardiomyocytes, as well as the effective prevention of DOX-induced cardiomyopathy [112].

### 5.4. Terpenoids

Terpenes are plant substances found in herbs and fruits. Terpenes are hydrocarbons, while terpenoids are terpenes containing an additional oxygen molecule. Terpenoids are found in abundance in natural settings and are known to influence the growth, death, and spread of cancer cells. Artemisinin is a compound primarily utilized for the treatment of malaria, although its derivatives have been shown to possess anticancer properties [113]. The results of studies conducted on rats have demonstrated the efficacy of intra-gastric administration of artemisinin in reducing hepatotoxicity and cardiotoxicity caused by DOX. A therapeutic dose of 7 mg/kg, as well as a significantly increased dose of 35 mg/kg, has been shown to be effective in attenuating these side effects. In particular, artemisinin has been shown to significantly decrease DOX-dependent caspase 3 and to increase TNF-α, iNOS, and NF-κB [100]. Oridonin exerts effective anti-tumor effects by regulating various intracellular signaling pathways. The combination of oridonin with DOX demonstrated a significant anti-tumor effect while reducing cardiotoxicity, according to Li. et al. [114]. This was achieved through the accumulation of the latter in breast cancer cells, which in turn induced apoptosis and inhibited the activity of vascular endothelial growth factor receptor 2 (VEGFR2), a key factor in angiogenesis. Interesting results were reported by Quagliariello et al., where nanoemulsions loaded with lycopene-rich tomato extract were investigated. In a DOX-induced cardiotoxicity in vitro model, H9C2 cells treated with nanoemulsion with DOX showed enhanced viability as well as decreased levels of the pro-inflammatory factors IL-6, IL-8, IL-1β, and TNF-α when compared to those treated with DOX alone [115].

The efficacy of limonin in preventing DOX cardiotoxicity has been demonstrated in laboratory trials, which have revealed the compound’s ability to activate the Nrf2 and Sirt2 signaling pathways [116]. In a study conducted by Meeran et al. [117], male albino rats were administered DOX in combination with oral nerolidol treatment (5 mg/kg) for 5 weeks. The results demonstrated that nerolidol exhibited a superior protective effect against chronic cardiotoxicity in DOX rats. In comparison to DOX alone, nerolidol demonstrated the capacity to reverse the increase in myocardial marker enzymes and hemodynamic changes caused by DOX. This was achieved by effectively activating the PI3K/Akt/Nrf2 pathway and inhibiting the NF-κB/MAPK pathway. Moreover, the results indicated that nerolidol had a positive impact on the oxidative damage caused by DOX and myocardial fibrosis, as well as on the regulation of inflammatory factors. 

### 5.5. Polysaccharides

In addition to saponins, the active substances of astragalus are polysaccharides. The presence of polysaccharides in the plant has been shown to stimulate the immune system and strengthen the body. Astragalus polysaccharide can suppress the generation of ROS in cardiomyocytes and the phosphorylation of p38MAPK by activating the PI3K/Akt signaling pathway. It can also inhibit ROS production and p38MAPK phosphorylation and mitigate the adverse effects of doxorubicin, including apoptosis, oxidative stress, and cellular inflammation [118]. A study demonstrated that administration of DOX in combination with astragalus polysaccharide (1.5 g/kg/day) for three days inhibited activation of the autophagy marker LC3B II/I, reduced caspase-3 expression, and promoted Bcl-2 protein expression [119]. The AMPK/mTOR signaling pathway, activated by astragalus polysaccharide, alleviates DOX-induced abnormal autophagic flow by preventing excessive apoptosis, thereby exerting a protective effect on myocardial injury. Liu et al. [120] demonstrated that astragalus polysaccharide microvesicles combined with ultrasound could activate the AMPK pathway, thereby increasing PPAR-γ expression and inhibiting NF-κB activity. This resulted in a significant reduction in oxidative stress and the inflammatory response of myocardial tissue, improvement in glucose tolerance and blood lipid levels, and effective alleviation of cardiomyopathy in diabetic rats. The administration of astragalus polysaccharide (1 g/kg/day) to DOX mice for three days by Zhang et al. [121] resulted in the establishment of new guidelines for the regulation of sphingomyelin and glycerophosphatidyl metabolic processes, which led to a significant reduction in interstitial edema and vacuolization of myocardial tissue. Furthermore, the guidelines improved cardiac dysfunction in mice.

## 6. Conclusions and Perspectives 

Doxorubicin has a well-established track record of efficacy in cancer treatment. However, there is still a challenge to be overcome regarding its use. This is the issue of cardiotoxicity. Various strategies have been explored in an attempt to address these limitations, with chemical modification being one option. While this can separate its pharmacological effects from its toxic side effects, it has the downside of reduced toxicity, which often comes at the cost of reduced efficacy. A potential and practical approach is therefore to combine drugs in order to achieve a better balance. 

The pathogenesis of doxorubicin-induced cardiomyopathy is multifactorial, encompassing a plethora of mechanisms. Given the multiplicity of mechanisms involved in doxorubicin-induced cardiotoxicity, it is difficult to identify a precise molecular target for toxicity. An interesting approach has been proposed by Amgalan et al., targeting BAX, which is a member of the BCL-2 protein family. Alterations of its conformation and activation during stress conditions result in promoting apoptosis and necrosis, so administration of small-molecule allosteric inhibitors of BAX may be efficient in the prevention of DOX-induced cardiotoxicity [122]. A study by Tedesco et al. suggests that supplementation of a mixture containing branched-chain amino acids with tricarboxylic acids (namely α5) may prevent DOX-induced cardiotoxicity via activation of the KLF15/Akt/eNOS/mTOR signaling axis [123]. Promising results were reported in the study by Quagliarello et al., where a combination of extracts of *Spirulina platensis*, * Ganoderma lucidum*, and * Moringa oleifera* (so-called Singo) was investigated. This complex nutraceutical formulation, rich in proteins, amino acids, polyphenols, terpenoids, and polysaccharides, significantly improves LVEF and radial/longitudinal strain in a DOX-induced cardiotoxicity mice model [124].

The findings of the literature review suggest that natural products may offer cardioprotective benefits against doxorubicin-induced cardiotoxicity, both in vitro and in vivo. However, further confirmatory studies are required to substantiate this claim. It is also notable that some of the reported effects do not compromise the anti-tumor efficacy of DOX. Nevertheless, the majority of these studies have focused on the impact of oxidation, inflammation, and apoptosis. It is of the utmost importance to direct greater attention towards the intricate signaling networks that are of paramount importance for the survival and dysfunction of cardiomyocytes. Moreover, the combination of DOX with natural products can result in reduced levels of DOX in healthy tissues, elevated levels of DOX in cancerous cell types, and an enhancement of the anticancer effects of DOX. 

Notwithstanding the encouraging progress made in the preclinical studies of natural products for the prevention of DOX-induced cardiotoxicity, these have not yet been translated for clinical use. One of the most significant obstacles hindering the development of cardioprotective adjuvants based on natural products is the lack of adequate bioavailability in humans. In consequence, the generation of synthetic derivatives or alternative methodologies is required in order to enhance the bioavailability of natural products. Moreover, the development of DOX-induced cardiotoxicity has been based on animal models and primary cultured cells. Consequently, future studies on the protective effects of DOX-induced cardiotoxicity should utilize newly developed models. Moreover, the identification of reliable clinical biomarkers of early myocardial changes that allow for the prediction of DOX-induced cardiotoxicity and the earliest possible intervention is of significant importance. 

## Figures and Tables

**Table 1:** Selected anthracycline mean equivalent doses *. Adopted from [15].

	Doxorubicin	Epirubicin	Daunorubicin	Idarubicin	Mitoxantrone
CVD dose ratio	1	0.8	0.6	NE	10.5

* Data derived from patients with a median age of 6.1 years and who survived at least five years after anthracycline therapy. NE—not estimated.

**Table 2:** DOX derivatives undergoing clinical trials.

Drug	Molecule	Clinical Trial Phase	Target/Effect	Ref.
Aldoxorubicin	Hydrazone derivative of doxorubicin	III	Relapsed/refractory, soft-tissue sarcoma; The results of the phase III study did not demonstrate a beneficial effect on median progression-free survival or median overall survival in the entire patient population.	[68]
Camsirubicin	13-deoxy-5-imino analogue of doxorubicin	II	Soft-tissue sarcoma; Significant myelosuppression	[69]
Annamycin	Iodine sugar derivative and liposomal formulation	I/II	Soft-tissue sarcoma, acute myelogenous leukemia, pancreatin carcinoma; 30-fold enrichment in lungs targeting; no cardiotoxicity (FDA-certified)	[69]
DTS-201	tetrapeptide pro-drug	I	Solid tumors	[69]

**Table 3:** Protective effect of polyphenols against antracycline-induced cardiotoxicity.

Compound	Model	Result	Mechanism	References
Eugenol	Sprague Dawley rats	Improved myocardial injury and protected heart function.	Decreased myocardial oxidative stress and Ca^2+^ accumulation.	[94]
6-gingerol	Albino rats	Improved myocardial injury.	Decreased myocardial oxidative stress and inhibited apoptosis.	[95]
Hesperetin	Wistar rats	Improved myocardial injury and apoptosis.	Decreased myocardial oxidative stress and DNA damage.	[96]
Isorhamnetin	Sprague Dawley rats H9c2 cells	Improved myocardial injury and histopathological damage.	Decreased myocardial oxidative stress and inhibited mitochondrial dysfunction-mediated cardiomyocyte apoptosis.	[97]
Kaempferol	Sprague Dawley rats H9c2 cells	Improved myocardial injury, improved body weight, heart weight, survival rate and cardiac function_._	Inhibited activation of p53-mediated, mitochondrion-dependent apoptotic pathway.	[98]
Salvianolic acids	Kunming mice	Improved myocardial injury and histopathological damage, protected heart function.	Decreased myocardial oxidative stress.	[99]
Silybinin	Wistar rats microsomes and mitochondria	Improved myocardial, liver injury, histopathological and heart membrane damage, protected heart and liver function.	Not mentioned.	[100]
Silychristin	Wistar rats microsomes and mitochondria	Improved heart membrane damage.	Decreased myocardial oxidative stress.	[100]
Silydianin	Wistar rats microsomes and mitochondria	Improved heart membrane damage.	Decreased myocardial oxidative stress.	[100]

## Abbreviations

ABC	ATP-binding cassette
ACEIs	angiotensin-converting enzyme inhibitors
AE	adverse effects
AIC	anthracycline-induced cardiotoxicity
ARBs	angiotensin receptor blockers
BMI	body mass index
BNP	B-type brain natriuretic peptide
BP	blood pressure
CABG	coronary artery bypass graft
CAT	catalase
CK	creatine kinase
CKD	chronic kidney disease
CK-MB	creatine kinase-MB
cTn	cardiac troponin
CTRCD	cancer therapy-related cardiac dysfunction
CV	cardiovascular
CVD	cardiovascular disease
DM	diabetes mellitus
DOX	doxorubicin
eNOS	endothelial nitric oxide synthase
ESC	European Society of Cardiology
FDA	Food and Drug Administration
Ft	ferritin
GRP78	glucose-regulated protein 78
HF	heart failure
HFA	Heart Failure Association
HT	hypertension
ICOS	International Cardio-Oncology Society
IL-1β	interleukin-1β
IL-6	interleukin-6
iNOS	induced nitric oxide synthase
IRPs	iron regulatory proteins
KLF15	Krüppel-like factor 15
LDH	lactate dehydrogenase
LV	left ventricular
LVEF	left ventricular ejection fraction
MAPK	mitogen-activated protein kinase
MDA	lipid peroxidation marker
MI	myocardial infarction
MMP	matrix metalloproteinase
MnSOD	mitochondrial superoxide dismutase
NADH	nicotinamide adenine dinucleotide
NADPHNOX2	nicotinamide adenine dinucleotide phosphateNADPH oxidase 2
NF-κB	nuclear factor kappa-light-chain-enhancer of activated B cells
NLPR3	nucleotide-binding domain-like receptor protein 3
NP	natriuretic peptides
Nrf2	nuclear factor-erythroid 2-related factor 2
PCI	percutaneous coronary intervention
ROS	reactive oxygen species
RT	radiotherapy
SGLT-2	sodium glucose transporter protein 2
SLC	solute carrier transporter
SNPs	single nucleotide polymorphisms
SOD	superoxide dismutase
TIF1	transcription intermediary factor 1
TLR	toll-like receptor
TNF-α	tumor necrosis factor-α
VEGFR2	vascular endothelial growth factor receptor 2
VHD	valvular heart disease
WGS	whole genome sequencing
